# Oriented artificial nanofibers and laser induced periodic surface structures as substrates for Schwann cells alignment

**DOI:** 10.12688/openreseurope.17370.3

**Published:** 2024-10-22

**Authors:** Sebastian Lifka, Cristina Plamadeala, Agnes Weth, Johannes Heitz, Werner Baumgartner

**Affiliations:** 1 Institute of Biomedical Mechatronics, Johannes Kepler University Linz, Linz, Upper Austria, 4040, Austria; 2 Institute of Applied Physics, Johannes Kepler University Linz, Linz, Upper Austria, 4040, Austria

**Keywords:** Aligned Nanofibers, Electrospinning, Nanoripples, Laser-Induced Periodic Surface Structures, Nerve Regeneration, Tissue Engineering, Schwann cells

## Abstract

People with injuries to the peripheral nervous system suffer from paralysis of the facial muscles, fingers and hands or toes and feet, often for the rest of their lives, due to its poor functional regeneration. Therefore, to improve patients’ quality of life, there is an urgent need for conduits that effectively support the healing of large defects in nerve pathways through specific guidance of nerve cells. This paper describes two specific methods for achieving directed growth of Schwann cells, a type of glial cells that can support the regeneration of the nerve pathway by guiding the neuronal axons in the direction of their alignment. One method uses aligned polyamide-6 (PA-6) nanofibers produced via electrospinning on a very fast rotating structured collector, which enables easy nanofiber detachment, without additional effort. The other method implies the exposure of a poly(ethylene terephthalate) (PET) foil to a KrF* laser beam, that renders a nanorippled surface topography. Schwann cell growth on these substrates was inspected after one week of cultivation by means of scanning electron microscopy (SEM). For both methods we show that Schwann cells grow in a certain direction, predetermined by nanofiber and nanoripple orientation. In contrast, cells cultivated on randomly oriented nanofibers or unstructured surfaces, show an omnidirectional growth behavior. These two methods can be used to produce nerve conduits for the treatment of injuries to the peripheral nervous system.

## Introduction

In the peripheral nervous system of vertebrates, neurons are responsible for receiving and transmitting electrical and chemical signals and glial cells provide the necessary support and protection for neurons. Here, the most important glial cells are Schwann cells. They are responsible for the creation of myelin sheaths that protect and insulate neuronal axons ( Cattin *et al*., 2015). They are also essential and indispensable for axon regeneration in the event of injury ( Manganas *et al.*, 2022). The peripheral axons regenerate in regeneration tracks (i.e., bands of Büngner) formed by elongated Schwann cells ( Jessen & Mirsky, 2019). Small gaps can regenerate on their own, while large gaps are considered to require the support of a nerve graft.

Today’s gold standard in clinical treatment of nerve injuries are autografts for bridging large gaps. However, limitations like donor site morbidity and the need of a second surgery leave room for improvement. An alternative to nerve autografts are nerve allografts. Although these avoid the disadvantages of autografts mentioned above, they require immunotherapy for approximately 18 months to prevent rejection of the graft ( Meena *et al.*, 2021). A third possibility for clinical treatment of large nerve injuries are artificial nerve conduits. Current commercially available conduits consist of simple hollow tubes connecting the two damaged nerve stumps and aim for increasing the number, speed, and length of the regenerating axons ( Meena *et al.*, 2021). These conduits must exhibit adequate properties like biocompatibility, biodegradability, mechanical properties, and biochemical properties. The major challenges of nerve conduits are wrong target innervations and axonal dispersions, for example. Furthermore, their repair length is currently limited to less than 3 cm in humans ( Meena *et al.*, 2021).

However, the currently available artificial nerve grafts have insufficient success rates, especially for large nerve gaps of several centimeter. A key factor here is the elongated orientation of the Schwann cells which is induced by stimuli from their surrounding either in the extra cellular matrix (ECM) or in case of artificial implants by the supporting material ( Jiang *et al.*, 2024; Manganas *et al.*, 2022).

One possibility to guide Schwann cells would be the use of natural silk. ( Millesi *et al.*, 2021) and ( Stadlmayr *et al.*, 2024), for example, have recently conducted promising research into the use of natural spider silk in nerve regeneration. Since the harvesting of natural silk can be costly and time-consuming, the use of artificial fibers, such as nanofibers produced using the so-called electrospinning process, would be advantageous. As nanofiber materials for nerve guidance conduits, one could employ electrospun fibers from materials that are biocompatible and biodegradable (i.e., poly-ε-caprolactone or collagen/poly-ε-caprolactone blends). Results of an earlier activity in this direction are reported in ( Schnell *et al.*, 2007). In the process of electrospinning, the fibers became oriented more or less parallel to each other as a suspended array between the two electrode bars. Under optimized conditions, for instance deposition on a drum or disc electrode with high-speed rotation ( Chen *et al.*, 2021; Wang *et al.*, 2018), it is possible to produce, nanofiber mats with parallel orientation. Directed nanofibers have also been used as scaffolds in nerve regeneration. ( Huang *et al.*, 2015) used directed cellulose acetate butyrate (CAB) nanofibers for the regeneration of nerves in rats. However, the removal of the nanofiber non-woven from the electrodes or supporting materials without tearing remains problematic. To fill conduits with a larger lumen with parallel oriented fibers, the combination of 3D frames (i.e., a stair case like target) and supporting gels have already been tested in animal experiments ( Kriebel *et al.*, 2017). Also, roll-up memory shape supports were considered ( Wang *et al.*, 2020).

A second possibility to guide Schwann cells is to create a substrate with nanoripples, also known as laser-induced periodic surface structures (LIPSS), that can be achieved by short-pulsed laser processing ( Bonse *et al.*, 2017). In previous work, we and others have investigated the effects of laser-induced nanostructures and hierarchical micro/nanostructures on adherent cells cultured thereon ( Fosodeder *et al.*, 2020; Maalouf *et al.*, 2022; Muck *et al.*, 2021). Depending on the specific structure, cell adhesion, proliferation, or migration can be inhibited, cell alignment can be induced for alignment, production of extra-cellular matrix proteins can be promoted, or the differentiation of stem cells into specific cell types can be supported. It has been shown, that laser-induced nanoripples and dual-rough hierarchical micro/nanostructures can be used to control Schwann cells attachment and migration, at least on silicon ( Yiannakou *et al.*, 2017). However, to our knowledge no detailed investigations were performed so far with Schwann cells on laser-structured polymeric materials, which could be of relevance for nerve implants.

In this work we present two methods which can be used effectively in the future for the production of implants in nerve regeneration. One method enables the production of aligned nanofibers which can be easily detached from the electrospinning collector as an independent non-woven without additional coating or complex additional work, as it was the case up to now and thus significantly simplifies the manufacturing process. This is achieved by a collector rotating at a very high speed which winds up the electrospun fiber as if on a wire drum. The surface of the collector is structured with a special micro-surface structure, which has already been described in more detail in a previous publication ( Lifka *et al.*, 2023). The surface structure allows the nanofiber non-woven to be removed from the collector easily and without leaving any residue. This supportless electrospinning technique makes a simple and easy production of nerve guides possible and is therefore the unique feature of this method. The other method describes the production of LIPSS, also known as nanoripples, which, due to their resemblance to the ECM, enable the aligned growth of nerve cells. This method is, however, limited to more or less rigid polymer foils ( Plamadeala *et al.*, 2023; Rebollar *et al.*, 2008; Rebollar *et al.*, 2012) and is therefore only suitable to a limited extent for flexible nerve conduits. There are a few approaches to produce laser-induced nanoripples on very thin flexible polymer films ( Buchberger *et al.*, 2024) or on bendable polymer microfibers ( Sajzew *et al.*, 2015), but these are very delicate to handle. Therefore, the preferred method for the production of nerve conduits tends to be the method that uses nanofibers, as these have a high degree of flexibility. Nevertheless, we show for both methods that, in contrast to randomly oriented fibers or unstructured, flat surfaces, they promote oriented cell growth in a direction predefined by the orientation of the fibers and ripples. Both methods can be used for nerve conduit production, whether it is a surface structured polymer foil rolled up to a hollow tube, a thin and very flexible aligned nanofiber non-woven rolled up to a thin roll, or a combination of both, hence, a nanofiber roll wrapped in a thin PET film.

## Methods

### Aligned nanofibers

The most important and novel part of this method is the rotating collector whose surface contains a structure in the µm range. This structure has already been presented in a previous publication and consists in principle of a simple triangular geometry which forms a jagged surface structure. In ( Lifka *et al.*, 2023), this structure was produced on a cylindrical collector with the aid of a thread turning tool in the form of a very fine thread, whereby the direction of preference of the structure is orthogonal to the collector axis. This variant represents the most unfavorable alignment of the structure for this application (the aligned nanofibers must be orthogonal to the direction of the structure in order to guarantee its function). Therefore, the structure must be manufactured in such a way that its orientation is parallel to the cylinder/collector axis. In the sense of simple, fast and cost-effective production, the triangular structure was produced by knurling the cylinder surface with axis-parallel grooves.

For this purpose, a cylindrical collector made of aluminum with a diameter of 40 mm was manufactured and machined on a lathe using a knurling wheel (ZEUS knurling wheel 11 AA 15 x 4 x 4 G7 T=0.3 PM a=90°, Article number: 41013392, Hommel+Keller Präzisionswerkzeuge GmbH, Aldingen, Germany) with the triangular surface structure. The structure therefore has a periodicity and depth of approximately 300 µm with a tip angle of 90°. To enable the collector to rotate, it is supported by two ball bearings (6001-2Z, SKF Österreich AG, Steyr, Austria) to the left and right of the structured surface. The bearing blocks were manufactured using a 3D printer (Photon Mono-X, Hongkong Anycubic Technology Co., Shenzen, China). The resin used for the 3D printed parts was the “Blu” resin from Siraya Tech (Blu-Tough Resin/Nylon Black, Siraya Tech, San Gabriel, California, USA). The collector is driven by a brushless DC motor (DB28M01, Nanotec Electronic GmbH & Co. KG, Feldkirchen, Germany) via a claw coupling (MJC19-4-A, JD12/19-85B; Ruland Manufacturing Co., Inc., Marlborough, UK). The maximum achievable rotational speed of the collector is approximately 14 meter per second. All CAD data and 2D drawing derivation required for the production of the collector are freely available in a Zenodo repository ( Lifka *et al.*, 2024).

**Figure 1.**
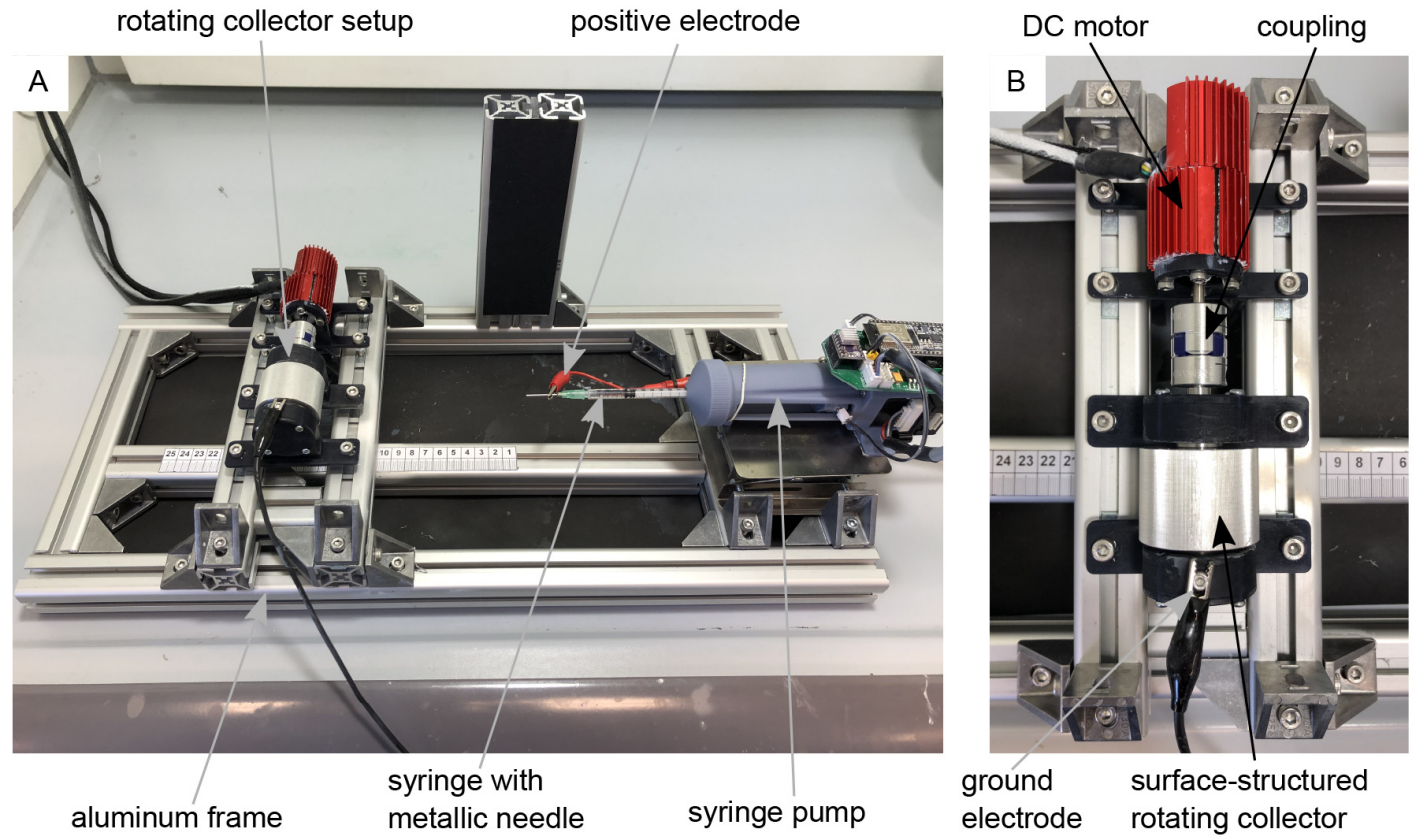
Electrospinning-setup for the production of aligned nanofibers. ( **A**) Custom-made electrospinning setup. ( **B**) Rotating drum collector setup mounted on the electrospinning setup-.

For electrospinning, the rotating drum collector assembly ( Figure 1B) was mounted on a custom-made electrospinning setup, as shown in Figure 1A. The setup is based on the classic horizontal electrospinning process in which a polymer solution is conveyed evenly through a thin metal needle with the aid of a syringe pump and applied to a collector in the form of thin nanofibers with the use of a strong electric field. The setup consists of a basic frame made of aluminum profiles which enables precise adjustment of the needle-collector distance. The polymer solution is conveyed evenly through a thin metal needle (Sterican 21G x 7/8″ blunt, B. Braun SE, Melsungen, Germany) using a custom-made syringe pump. The necessary electric field is generated by a high-voltage generator (HCP 35-35,000, FuG Elektronik GmbH, Schechen, Germany). The needle is the positive electrode and the collector is the negative electrode. The needle is contacted directly via a crocodile clip, while the collector is contacted via the conductive ball bearings to avoid sliding contact. The polymer solution for the nanofibers consists of 15 wt.% polyamide-6 (PA-6) (provided by Elmarco s.r.o., Liberec, Czech Republic) dissolved in formic acid (FA) (ROTIPURAN® ≥98%, p.a., ACS, Art. No. 4724.1, Carl Roth GmbH + Co. KG., Karlsruhe, Germany), and acetic acid (AA) (ROTIPURAN 100%, p.a., Art. No. 3738.4, Carl Roth GmbH + Co. KG., Karlsruhe, Germany) in a (weight) ratio of 2:1. A total of 15 g of the polymer solution was prepared, for which 2.25 g PA-6, 4.25 g FA and 8.5 g AA were used. The parameters for the spinning process were selected as follows: Needle-collector distance of 13 cm, needle-collector voltage of 20 kV, and flow rate of 0.34 mLh ^-1^.

The motor speed was controlled and set via the software “ Plug & Drive Studio 3” (v. 1.5.3.0, Nanotec Electronic GmbH & Co. KG, Feldkirchen, Germany). Various rotational speeds of the collector were tested until the fibers were oriented in the desired direction. The electrospinning process took about 10 minutes for each sample. After this time, a fleece that was thick and robust enough for further processing could be guaranteed. The finished non-woven was then cut open with a scalpel parallel to the collector axis and carefully removed from the collector with tweezers. Thanks to the surface structuring, the fleece could be removed easily and without leaving any residue. The Zenodo repository ( Lifka *et al.*, 2024) contains a video (Non-Woven_Detachement_from_Collector.mkv) showing the removal process of the aligned nanofiber non-woven. The specimens of the nanofiber non-woven were then sputter-coated with gold (SCD 005, BAL-TEC Inc., Balzers, Liechtenstein) for 80 s with 21 mA and examined under a scanning electron microscope (SEM) (Philips 525, Philips Electron Optics, Amsterdam, The Netherlands). The SEM images subsequently serve as the basis for the analysis of the directionality and the average fiber diameter.

The statistical analyses of fiber directionality and fiber diameter were performed using ImageJ (v. 1.51w, Rasband, W.S., U. S. National Institutes of Health, Bethesda, Maryland, USA). The directionality analysis function (v2.3.0, method: Fourier components) was used to analyze the fiber orientation. The DiameterJ (v. 1-018) plug-in ( Hotaling *et al.*, 2015) was used to analyze the fiber diameter. The corresponding diagrams were created with the help of Python-3 (v. 3.10.9, Python Software Foundation, Beaverton, Oregon, USA) and matplotlib (v. 3.3) ( Hunter, 2007) from the .csv files generated by ImageJ. All underlying data can be found in the Zenodo repository ( Lifka *et al.*, 2024).

### Nanoripples

**Figure 2.**
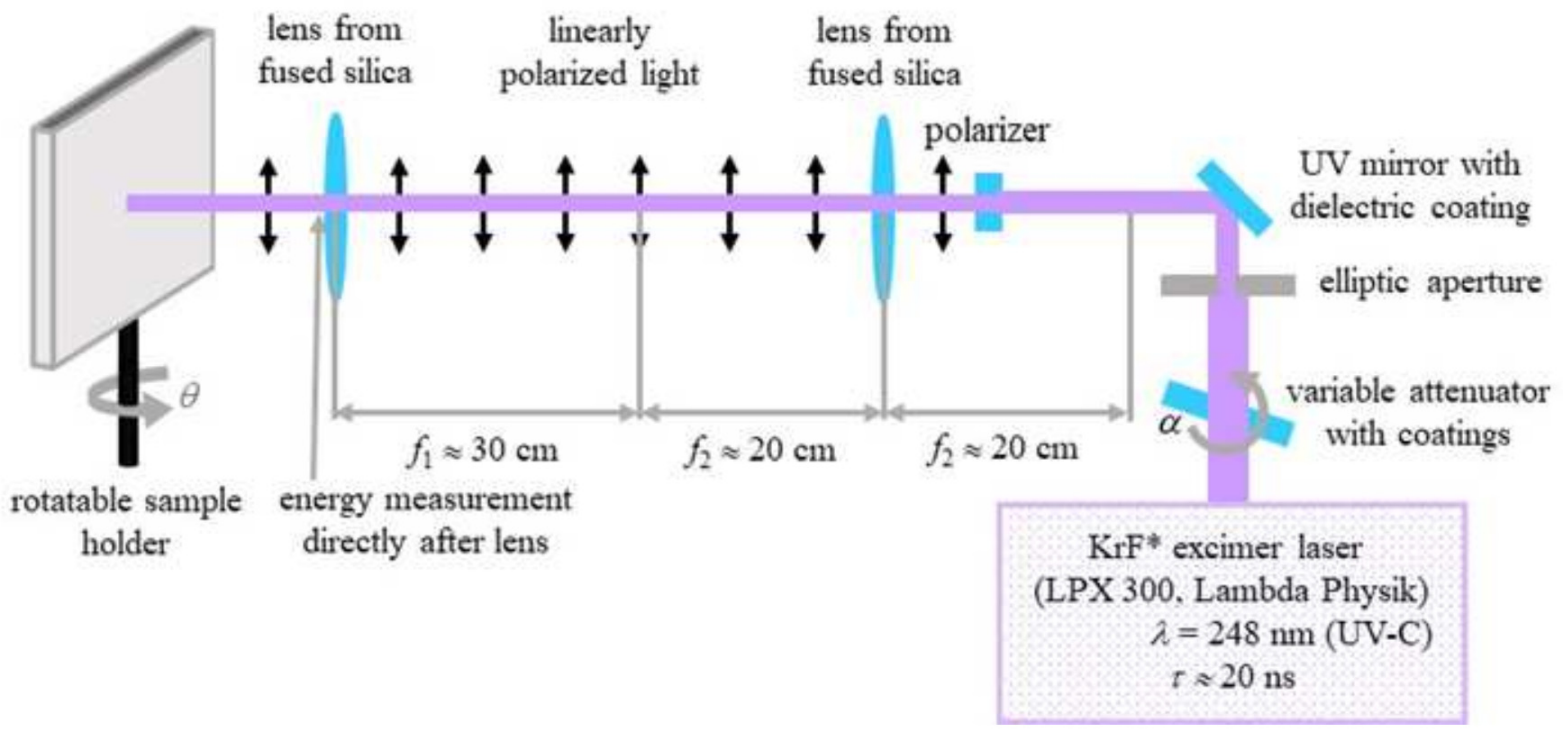
Setup for laser-induced periodic surface structures fabrication on poly(ethylene terephthalate) foils. (Reprinted from ( Buchberger *et al.*, 2023) which is an open-access article distributed under the terms of the Creative Commons Attribution 4.0 International (CC-BY 4.0)).

To produce ripples on poly(ethylene terephthalate) (PET) foils with a thickness of 50 µm (Goodfellow Ltd., Bad Nauheim, Germany), a KrF* (krypton fluoride) excimer laser (LPX 300, Lambda Physik, 181 Göttingen, Germany) was used, with a wavelength of 248 nm, 20 ns pulse duration, and 10 Hz pulse repetition rate. The setup is similar to the setup for laser processing of PET foils used in ( Richter *et al.*, 2021) and is shown in Figure 2. Prior to exposure, the PET foils were rinsed with ethanol and thoroughly dried with nitrogen, in order to remove any debris. Later on, the foils were fixed onto the sample holder at a 30° incidence angle and exposed to 6000 pulses at an energy of 12.6 – 12.8 mJ, applying an average fluence of 10 mJ cm ^-2^. Laser beam energy was measured with a high-area high-damage energy sensor (J45LP-MUV, Coherent, Portland, United States) with the help of a laser energy meter (FieldMaxII-P™, Coherent, Portland, USA).

Scanning electron microscope (model REM 1540XB-Crossbeam, Zeiss, Oberkochen, Germany) images of nanoripples were acquired at ten different positions within the nanostructured area and analyzed by using the free software Gwyddion (v. 2.61, Czech _Metrology Institute, Brno, Czech Republic) in order to calculate the spatial period. The resulting average spatial period is *Λ* = 331 ± 11 nm. The underlying data for spatial period calculation “Spatial period calculation.docx“ can be found in the Zenodo repository ( Lifka *et al.*, 2024). The height of the resulting nanoripples was determined in previous works to be approximately between 110 nm to 160 nm ( Buchberger *et al.*, 2023; Heitz *et al.*, 2024).

### Cell cultivation

To validate and show the oriented cell growth on samples produced with the above described methods, murine Schwann cells (Immortalized Mouse Schwann Cells (IMS32), T0295, Applied Biological Materials Inc., Richmond, Canada) were cultured for ten days in 4 mL grow medium consisting of PriGrow III (TM003, Applied Biological Materials Inc., Richmond, Canada) + 10% fetal bovine serum (FBS)(F7524, Sigma-Aldrich, St. Lois, Missouri, USA) + 1% Penicillin/Streptomycin Solution (G255, Applied Biological Materials Inc., Richmond, Canada) at 37.0 °C and 5% CO _2_ in an incubator (New Brunswick Galaxy ^®^ 48 S CO2, Eppendorf, Hamburg, Germany) on the corresponding sample. The medium was changed every two days. Prior to cell cultivation, samples were coated with a poly-L-lysine (PLL) hydrobromide (P9155, Sigma Aldrich, Sigma-Aldrich, St. Lois, Missouri, USA) solution at a concentration of 40 µg ml ^-1^ in double-distilled water, incubated for 10 minutes at room temperature, then washed with distilled water, and finally air-dried at room temperature. The cell density upon seeding on the samples was 25,000 cells per cm ^2^ resulting in a total number of approximately 65,000 cells per sample.

### Image manipulation

**Figure 3.**
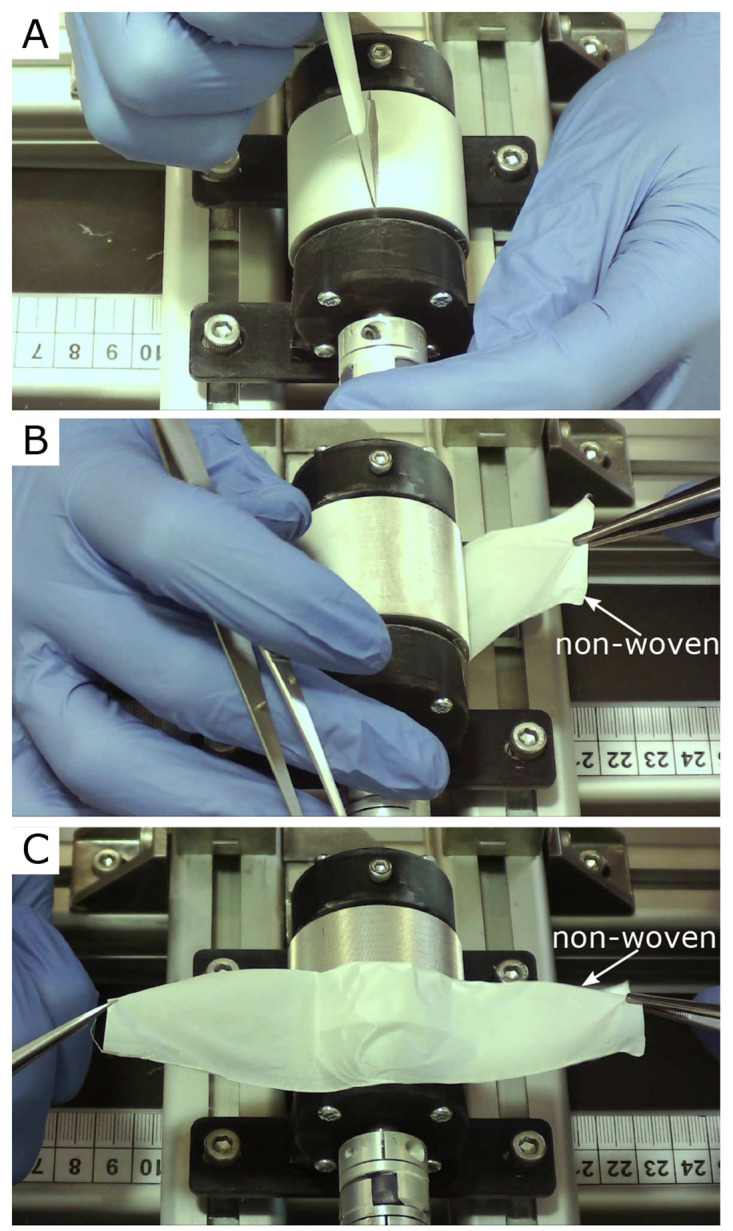
Removal process of the oriented nanofiber non-woven. ( **A**) Cut open the fleece with a scalpel. ( **B**) Remove the fleece with tweezers. ( **C**) Fleece completely detached from the collector without leaving any residue on the collector.

**Figure 4.**
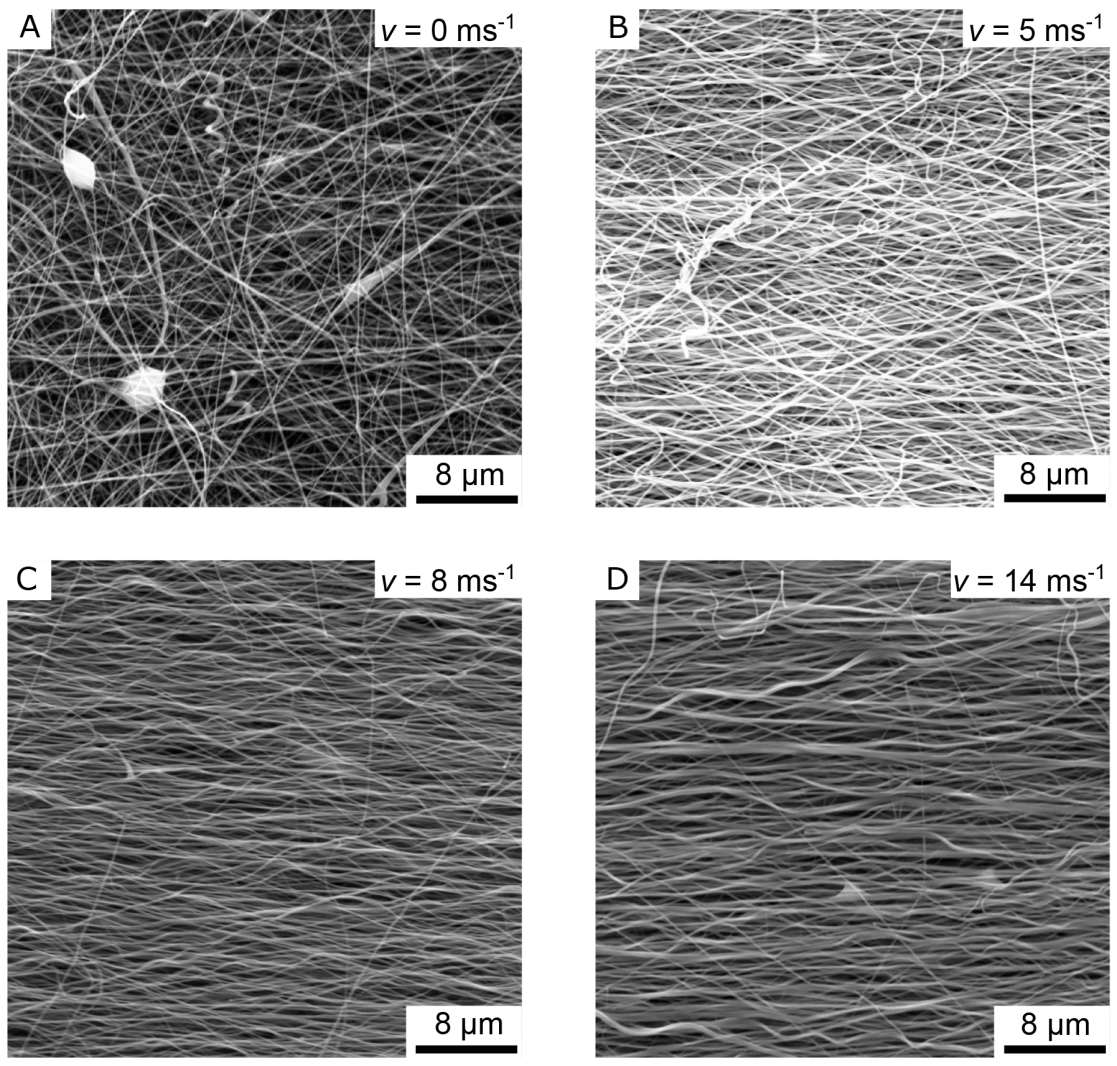
SEM images of the aligned nanofiber non-woven. ( **A**) Randomly oriented nanofibers as control (i.e., the collector did not rotate, *v* = 0 ms ^-1^). ( **B**) Nanofibers collected on the collector rotating with *v* = 5 ms ^-1^. ( **C**) Nanofibers collected on the collector rotating with *v* = 8 ms ^-1^. ( **D**) Nanofibers collected on the collector rotating with *v* = 14 ms ^-1^.

All images shown in the figures have been slightly manipulated to improve the clarity of the figures. The original, unmanipulated images can be found as underlying data in the Zenodo repository ( Lifka *et al.*, 2024). The software used for image manipulation was on the one hand Corel Vector (v. 23.0.0.363, Alludo, Ottawa, Canada) and on the other hand Inkscape (v. 0.92.4, The Inkscape Project c/o Software Freedom Conservancy, Brooklyn, New York, USA). While Corel Vector is a proprietary software, Inkscape is a freely available software that performs essentially the same functions. All image manipulations are listed in detail below:

- In Figure 1 the images “Figure 1A_original.jpg” and “Figure 1B_original.jpg” from the Zenodo repository were merged, labeled, and annotated in one Figure using Inkscape.
- In Figure 3 three single frames of the video “Non-Woven_Detachement_from_Collector.mkv” in the Zenodo repository were captured using the VLC media player (v. 3.0.20, VideoLan Organization, Paris, France), merged into one figure, labeled, and annotated using Inkscape.
- In Figure 4 the images “Figure 4A_original.tif”, “Figure 4B_original.tif”, “Figure 4C_original.tif”, and “Figure 4D_original.tif” from the Zenodo repository were merged, cropped, labeled, brightness and contrast optimized, and annotated in one Figure using Inkscape.
- In Figure 6 the images “Figure 6A_original.tif”, “Figure 6B_original.tif”, “Figure 6C_original.tif”, and “Figure 6D_original.tif” from the Zenodo repository were merged, labeled, rotated, brightness and contrast optimized, and annotated in one Figure using Inkscape.
- In Figure 7 the images “Figure 7A_original.tif”, “Figure 7B_original.tif”, “Figure 7C_original.tif”, and “Figure 7D_original.tif” from the Zenodo repository were merged, cropped, labeled, rotated, brightness and contrast optimized, and annotated in one Figure using Inkscape.
- In the Figure S1 embedded in the underlaying data “Spatial period calculation.docx” the image “Figure S1A_original.tif” from the Zenodo repository was labeled and annotated using Corel Vector.
- In Figure S3 the images “Figure S3A_original.tif”, “Figure S3B_original.tif”, and “Figure S3C_original.tif” from the Zenodo repository were merged, cropped, labeled, brightness and contrast optimized, and annotated in one Figure using Inkscape.

## Results

### Aligned nanofibers

Figure 3A to C show an example of the detachment process for the nanofiber non-woven. It can be seen that the non-woven can be removed easily and without leaving any residue on the collector. The video “Non-Woven_Detachement_from_Collector.mkv” in the Zenodo repository ( Lifka *et al.*, 2024) shows the removal process again in detail.

Figure 4 shows the SEM images of four different nanofiber non-woven samples. Figure 4A shows a non-woven with randomly oriented fibers (i.e. the collector did not rotate) as a control. Figure 4B to C show non-woven with increasing collector rotation speed. It can be clearly seen that the degree of orientation of the fibers increases with the rotational speed of the collector. While the orientation of the fibers still has potential for improvement at a rotational speed of *v* = 5 ms ^-1^ ( Figure 4B), a clear orientation in the horizontal direction can already be seen at a rotational speed of *v* = 8 ms ^-1^ or *v* =14 ms ^-1^ ( Figure 4C and D).

**Figure 5.**
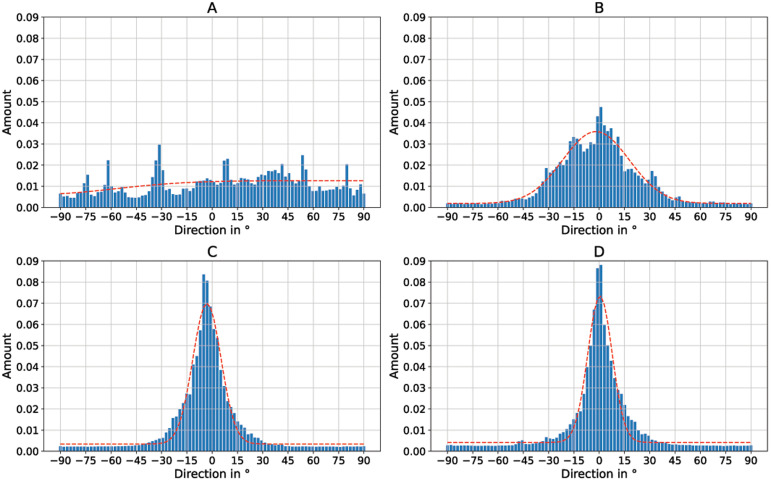
Directionality histograms of the non-woven. ( **A**) Randomly oriented nanofibers as control (i.e., the collector did not rotate, *v* = 0 ms ^-1^, *σ* = 31.08°). ( **B**) Nanofibers collected on the collector rotating with *v* = 5 ms ^-1^ ( *σ* = 19.68°). ( **C**) Nanofibers collected on the collector rotating with *v* = 8 ms ^-1^ ( *σ* = 8.37°). ( **D**) Nanofibers collected on the collector rotating with *v* = 14 ms ^-1^ ( *σ* = 7.24°). The dashed red line represents a normal distribution fit of the histogram.

To better quantify the directionality of the fibers, Figure 5 shows the directionality histograms for all SEM images of Figure 4. A horizontal line in the images corresponds to an angle of 0° in the histogram. The dashed curve in red represents a normal distribution fit. It can be clearly seen that the sample with the randomly oriented fibers ( Figure 5A) is relatively evenly distributed in all directions (standard deviation *σ* = 31.08°). The sample with *v* = 5 ms ^-1^ ( Figure 5B) is much more directional in comparison ( *σ* = 19.68°). The samples in which the collector was rotated at *v* = 8 ms ^-1^ ( Figure 5C) and *v* = 14 ms ^-1^ ( Figure 5D) both exhibit excellent directionality ( *σ* = 8.37° for 8 ms ^-1^ and *σ* = 7.24° for 14 ms ^-1^).

Table 1 lists the average fiber diameters of the individual samples. On average across all samples, the fibers produced had a diameter of approx. 250 nm with a standard deviation of approximately 110 nm.

**Table 1.** Mean fiber diameters of the samples.

	Mean fiber diameter in nm	Standard deviation in nm
**Randomly** **oriented**	277.5	110.4
**5 ms ^-1^**	205.9	81.3
**8 ms ^-1^**	226.5	101.4
**14 ms ^-1^**	309.0	151.5
**Overall**	254.725	111.15

**Figure 6.**
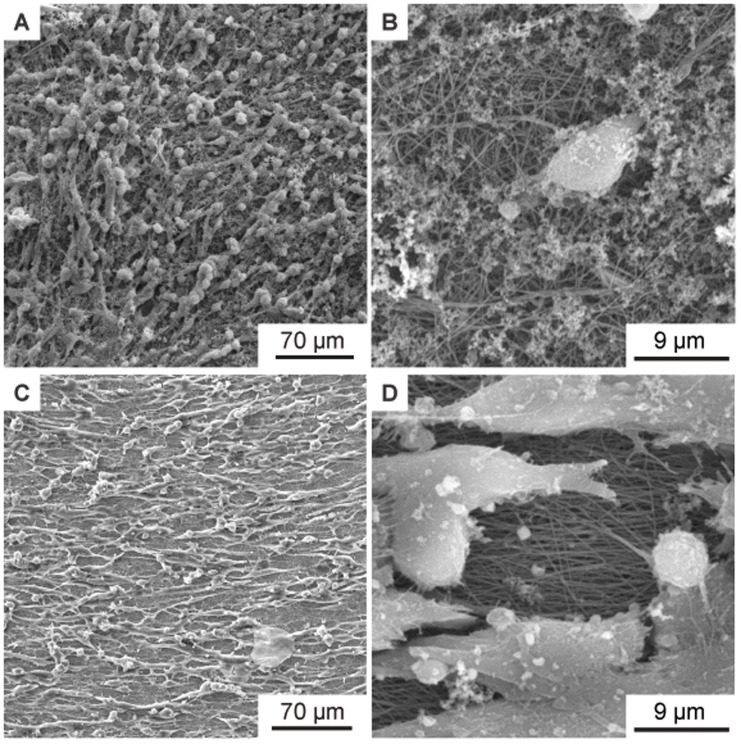
SEM images of murine Schwann cells cultivated for one week on electrospun PA-6 nanofibers. ( **A**) Cells on randomly oriented nanofibers showing no orientation as control. ( **B**) Magnified detail of ( **A**) showing the randomly oriented nanofibers under the cells. ( **C**) Cells with preferential orientation on aligned nanofibers (collector speed 8 ms-1). ( **D**) Magnified detail of ( **C**) showing the aligned nanofibers under the cells. In addition to the SEM images, Figure S3 in the Zenodo repository ( Lifka *et al.*, 2024) shows fluorescence microscopy images of the cells on the non-woven. Figure S3A shows randomly oriented cells on a flat cover glass as control, Figure S3B shows randomly oriented cells on randomly oriented nanofibers, and Figure S3C shows oriented cells on aligned nanofibers. A brief description of the staining method used can be found in the file “Description_Staining_Method.docx” in the Zenodo repository ( Lifka *et al.*, 2024).

To investigate the directional cell growth on the samples, murine Schwann cells were cultured on an 8 ms ^-1^ non-woven nanofiber sample ( Figure 4C), which has a parallel fiber direction ( Figure 5C) and on a sample with randomly oriented nanofibers as control. Despite the slightly smaller standard deviation of the 14 ms ^-1^ ( *σ* = 7.24°) sample in comparison to the 8 ms ^-1^ ( *σ* = 8.37°) sample, the 8 ms ^-1^ was preferred. This is due to the fact that a rotational speed of 14 ms ^-1^ pushes the limits of our setup and therefore the 8 ms ^-1^ sample is much easier to produce and that the standard deviations of the two samples are de facto identical. The results can be seen in Figure 6A (randomly oriented) and C (aligned). A clear orientation of the cell growth in a horizontal direction can be seen in Figure 6C. Figure 6B and D show an enlarged section of Figure 6A and C, in which the non-woven under the cells is also visible. Note that the orientation of the nanofibers is also horizontal (i.e., parallel to the direction of cell growth) in Figure 6D. Similar to Figure 5, the directionality of the cell growth was quantified using Fourier analysis. The respective directionality histograms for the cells on nanofibers are shown in Figure 8A (randomly oriented) and Figure 8B (aligned). The directionality is based on the long axis of the cells, in other words the long axis of the cells corresponds to 0° in the histogram. Additionally, the fibers under the cells were also analyzed using Fourier analysis. The respective directionality histograms are shown in Figure S2 as underlying data in the Zenodo repository ( Lifka *et al.*, 2024). The directional cell growth was investigated on *n* = 7 aligned 8 ms ^-1^ nanofiber samples in total. All samples showed directional growth of the Schwann cells in fiber direction as presented in Figure 6. Therefore, Figure 6 shows a typical result of one specific sample.

### Nanoripples

Nanoripples (LIPSS) are structures that form upon laser irradiation on many materials, and originate from the interference of the incoming linearly polarized light of (ultra-)short pulsed lasers with the radiation remnants or plasmon modes in the surface ( Bonse *et al.*, 2017). The orientation of the LIPSS can be either parallel or perpendicular to the polarization of the laser beam and their periodicity is comparable to the wavelength or smaller, depending on the laser fluence and the angle of incidence of the laser beam onto the irradiated substrate. In our case, the spatial period Λ of nanoripples fabricated using s-polarized laser light is given by *Λ* = *λ*/( *n _eff_* – sin *θ*), where *λ* is the wavelength of the laser beam, *n _eff_* – the effective refractive index which lies between the refractive indices of air and PET ( Barb *et al.*, 2014), and *θ* – the angle of incidence.

**Figure 7.**
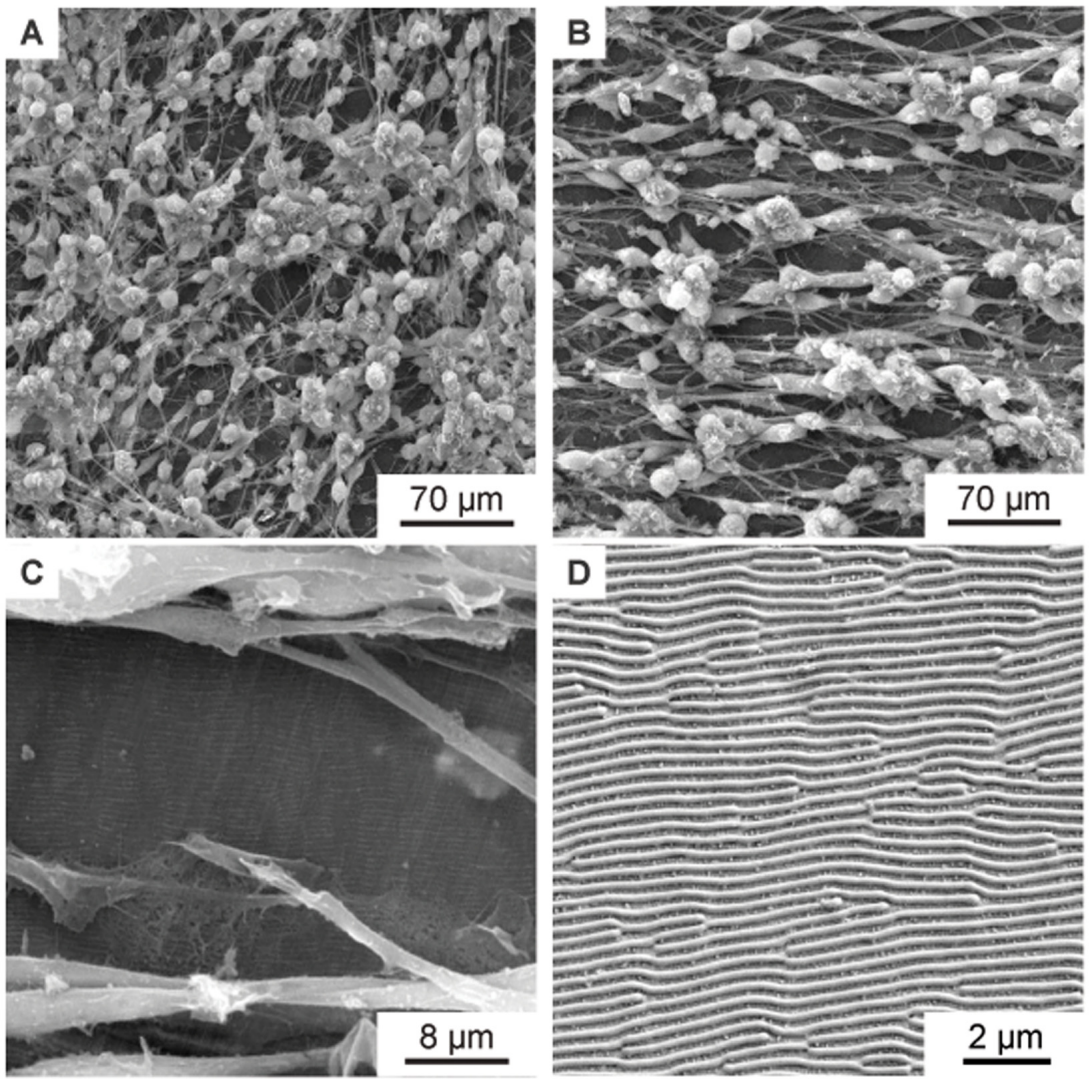
SEM images of aligned murine Schwann cells cultivated for one week on PET foils. ( **A**) SEM image of murine Schwann cells cultivated for one week on flat PET foil, as control, showing no alignment. ( **B**– **C**) SEM images of aligned murine Schwann cells cultivated for one week on a PET foil with nanoripples. ( **C**) is a magnification of ( **B**), showing an area with incomplete cell coverage, proving that the cells and the nanoripples have the same orientation. ( **D**) SEM image showing the nanoripples without cells.

**Figure 8.**
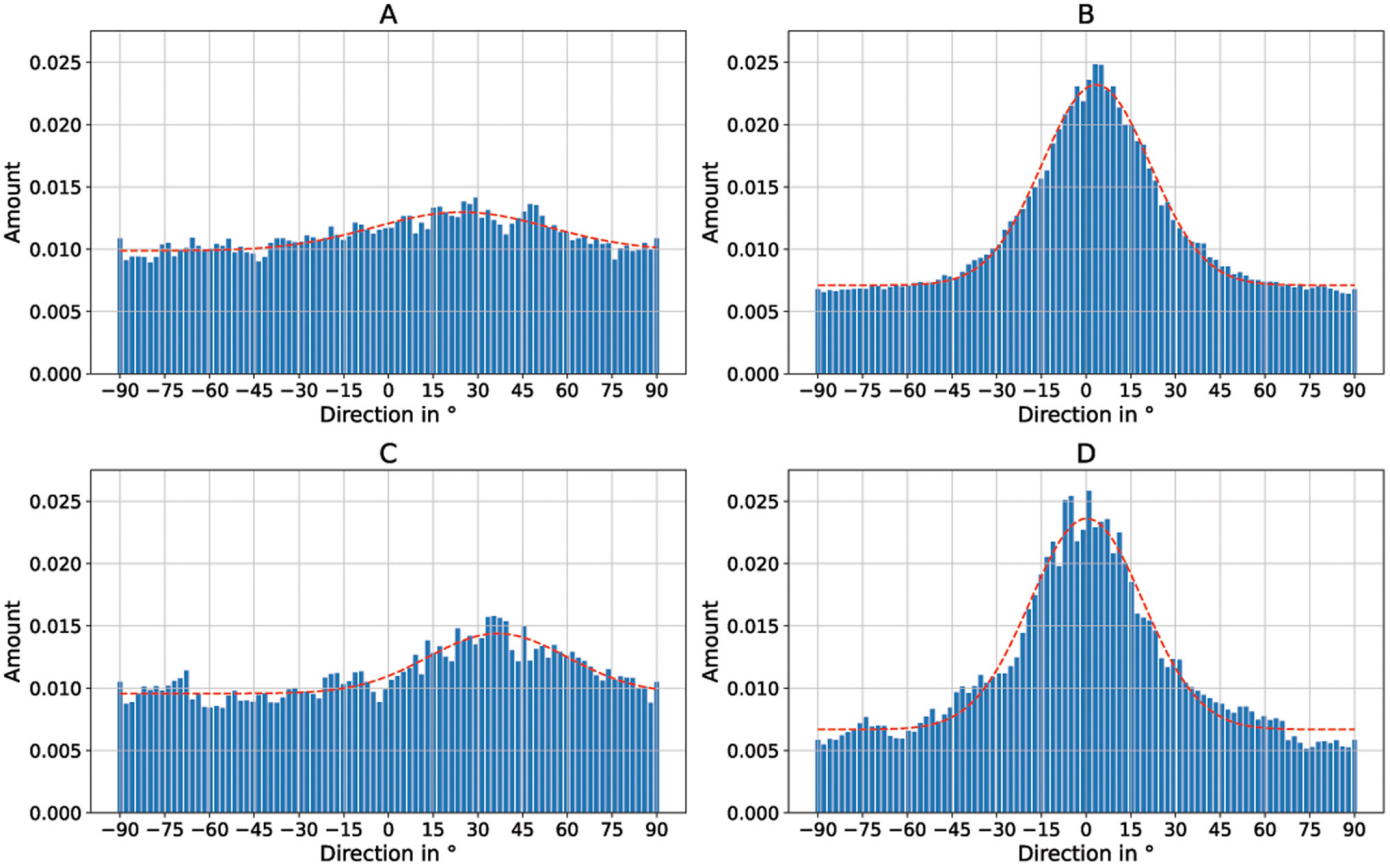
Directionality histograms of the murine Schwann cells cultivated on electrospun PA-6 nanofibers and PET foils. ( **A**) Cells on randomly oriented nanofibers ( *σ* = 29.51°). ( **B**) Cells on aligned nanofibers ( *σ* = 18.06°). ( **C**) Cells on flat PET foil ( *σ* = 23.37°). ( **D**) Cells on PET foil with nanoripples. ( *σ* = 18.95°). The dashed red line represents a normal distribution fit of the histogram.

Figure 7A shows the murine Schwann cells cultivated for one week (same procedure as described above) on flat PET foil, used as reference sample. The cells show a random orientation and omnidirectional growth. In contrast, Figure 7B, showing murine Schwann cells cultivated for one week on PET nanoripples, proves the influence of nanorippled topography as external stimuli that renders cell alignment. Schwann cells exhibit a typical elongated bipolar shape and even though their sizes (in the order of some tens of micrometers) are much bigger than the nanoripples’ height (above 110 nm ( Buchberger *et al.*, 2023)) and periodicity ( *Λ* = 331 ± 11 nm, *N* =10), the cells align and orient themselves along the LIPSS orientation. This effect happens due to the interaction of cell filopodia with the nano topography, as shown in Figure 7C. Figure 7D additionally shows the nanoripples without cells for better visibility. Again, the directionality histograms of the directed cell growth are shown in Figure 8C for the flat PET foil sample and in Figure 8D for the PET foil sample with nanoripples. Again, the long axis of the cells corresponds to 0° in the histogram.

## Discussion

In this work, two methods are described to present an improved directed growth of Schwann cells, a type of glial cell that insulates and protects the axons of neurons. Major injuries of nerve tissue are often characterized by poor functional regeneration and therefore require conduits that promote healing of the nerve axons and provide direction for Schwann cell growth. One method uses laser-induced periodic surface structures (LIPSS) that mimic the nanofeatures found in the extracellular matrix. These LIPSS can be used to specify the direction of growth for Schwann cells and promote growth in a specific direction. As we have reported before ( Barb *et al.*, 2014; Barb *et al.*, 2015; Rebollar *et al.*, 2008), different types of mammalian cells can be aligned by parallel quasi-periodic grooves or ripples on the underlying substrate even though the lateral dimensions of the cells are much larger than the structures as well as the height of the cells is much larger than the structure height. The lateral periodicities of the ripples or groove reach from a few hundred nm to a few µm with structure heights of a few ten to a few hundred nm, respectively. In this regime, which could be addressed as “surface-induced alignment”, the alignment of cells is based on organization of the fibrils of the cell cytoskeleton especially in protrusions of the cells ( Peterbauer *et al.*, 2010). For larger structure periodicities and structure heights, as for instance in ( Babaliari *et al.*, 2021), the structure dimensions become comparable to those of the cells and we see a “contact guidance” of the whole cells by the structure walls. When the oriented topographic features on a surface are too small, the cells cannot align along these structures any more. For laser-induced ripples, cell alignment is observed only when the periodicity is above a critical periodicity threshold (about 300 nm), which is cell type specific ( Rebollar *et al.*, 2008). For surface-induced alignment, we saw no qualitative difference in cell alignment depending on the structure size, at least not for human myoblasts (see ( Barb *et al.*, 2014; Barb *et al.*, 2015)). Both methods presented here provide parallel features which correspond to surface-induced alignment regime and we show here for the first time that they can be used for the orientation of Schwann cells, in deed. However, we expect no strong qualitative effect of a variation of the structure size (within this regime) on the alignment.

This is, however, limited to more or less rigid (in this case) PET films. Laser-induced nanoripples were reported on a number of other polymers, for instance polystyrene (PS) ( Rebollar *et al.*, 2008), poly (trimethylene terephthalate) and poly (carbonate bisphenol A) ( Rebollar *et al.*, 2012), poly(carbonate) (PC) ( Csete *et al.*, 2001; Mezera *et al.*, 2019), the epoxy resin SU-8 ( Plamadeala *et al.*, 2023), conjugated polymers ( Rodríguez-Rodríguez *et al.*, 2018), or poly(imide) (PI) ( Baudach *et al.*, 1999). These polymers have different material properties to PET to a certain extent, but are all fairly inflexible as they are several 10 µm thick sheets, rigid plates, or thin films on rigid substrates. They are therefore only suitable to a limited extent as nerve-conducting fillers in a flexible nerve regeneration conduit, but may be used as a hollow tube connecting two nerve ends in cases where extremely high flexibility is not absolutely necessary. There are a few approaches to produce laser-induced nanoripples on very thin flexible polymer films ( Buchberger *et al.*, 2024) or on bendable polymer microfibers ( Sajzew *et al.*, 2015), but these materials are very delicate to handle.

Therefore, an alternative method describes the production of a non-woven consisting of parallel, aligned nanofibers. This non-woven is extremely flexible and can be adapted to many different shapes. The method is based on the electrospinning process and uses a very fast rotating cylindrical collector to produce directed nanofibers. The unique feature of this method in comparison to existing techniques is the special surface structure ( Lifka *et al.*, 2023) of the collector, which enables the non-woven to be easily removed without additional coating and additional effort. (i.e., a supportless electrospinning process).

These extremely flexible non-woven nanofiber mats have huge potential for use in the manufacturing of 3D-conduits for nerve regeneration. The nanofiber sheets can easily be rolled up in the axial direction of the fibers. This results in a thin roll of nanofiber non-woven with the nanofiber orientation in axial direction. One or more of these rolls could now be used as a conduit for nerve regeneration by connecting the two ends of a damaged nerve pathway and thus simply bridging large defects. The aligned nanofibers now act as an orientation aid for the nerve cells, which can thus repair the defects more easily and quickly. Additionally, a thin laser-structured polymer foil can be wrapped around the nanofiber conduit in order to increase the stability of the whole conduit, which would combine the two presented methods in one application.

In the results presented here, the samples were coated with PLL to improve cell adhesion to the nanofibers. We observed that although the cells adhere without PLL coating, they immediately detach from the fibers at the slightest mechanical stress. For this reason, all experiments presented here were carried out with PLL-coated samples. For the sake of completeness, Figure S4 shows an SEM image of aligned Schwann cells on aligned nanofibers that were not PLL coated. Visually, there is no significant difference to PLL coated samples.

As shown in Table 1, the mean fiber diameter increases with the rotational speed. From Figure 5, it seems that the fiber shape changes to a more ribbon-like shape with increasing rotational speed, which may explain the increasing measured mean diameter. However, this phenomenon was not further investigated in the course of this work and is thus the subject of future research.

In this work, only one fiber material was tested, namely PA-6, as good experience has already been made with this polymer. Of course, this method can also be applied to biocompatible polymers, such as fibers made of cellulose acetate butyrate (CAB) or collagen fibers. These materials would be very well suited for use in nerve regeneration. The method presented here can easily be adapted for these materials. Only the relevant parameters, such as the rotational speed of the collector and the volume flow of the electrospinning solution, need to be determined and adapted accordingly.

## Conclusion

In this paper we show that oriented mechanical nanofeatures, such as electrospun aligned nanofibers and nanoripples produced by means of LIPSS on PET foils, are able to mimic the extracellular matrix features, and stimulate the elongation and support the alignment of Schwann cells. In particular, conduits made of flexible, rolled-up oriented nanofibers and hollow tubes of thin polymer foils with nanoripples could lead to better axonal regeneration in patients with severe nerve track injuries.

## Ethics and consent

Ethical approval and consent were not required.

## Data Availability

Zenodo: Repository for the Method Article "Oriented artificial nanofibers and laser induced periodic surface structures as substrates for Schwann cells alignment". https://doi.org/10.5281/zenodo.13944609 ( Lifka *et al.*, 2024). This project contains the following underlying data: Directionality_Hist.py. (Python file for generating directionality histograms of the non-woven.) RandomHist.csv. (Histogram data from ImageJ for randomly oriented non-woven.) 5mpsHist.csv. (Histogram data from ImageJ for 5 ms ^-1^ non-woven.) 8mpsHist.csv. (Histogram data from ImageJ for 8 ms ^-1^ non-woven.) 14mpsHist.csv. (Histogram data from ImageJ for 14 ms ^-1^ non-woven.) RandFibersHist.csv (Histogram data from ImageJ for cells on randomly oriented non-woven.) Fibers8mpsHist.csv (Histogram data from ImageJ for cells on 8 ms -1 non-woven.) RandFoilHist.csv (Histogram data from ImageJ for flat PET foil.) DirFoilHist.csv (Histogram data from ImageJ for PET foils with ripples.) RandFibersUnderCellsHist.csv (Histogram data from ImageJ for randomly oriented non-woven under the cells from Figure 6B.) Fibers8mps_FibersUnderCellsHist.csv (Histogram data from ImageJ for 8 ms -1 non-woven under the cells from Figure 6D.) DiameterAnalysisRandom.csv. (Fiber diameter analysis and histogram data from ImageJ for random oriented non-woven.) DiameterAnalysis5mps.csv. (Fiber diameter analysis and histogram data from ImageJ for 5 ms ^-1^ non-woven.) DiameterAnalysis8mps.csv. (Fiber diameter analysis and histogram data from ImageJ for 8 ms ^-1^ non-woven.) DiameterAnalysis14mps.csv. (Fiber diameter analysis and histogram data from ImageJ for 14 ms ^-1^ non-woven.) Spatial period calculation.docx. (Word file containing the data for the spatial period calculation of the nanoripples.) Description_Staining_Method.docx. (Word file containing a brief description of the staining method used for fluorescence microscopy.) Figure 1A_original.jpg. (Original unmanipulated image used for Figure 1A.) Figure 1B_original.jpg. (Original unmanipulated image used for Figure 1B.) Figure 4A_original.tif. (Original unmanipulated image used for Figure 4A.) Figure 4B_original.tif. (Original unmanipulated image used for Figure 4B.) Figure 4C_original.tif. (Original unmanipulated image used for Figure 4C.) Figure 4D_original.tif. (Original unmanipulated image used for Figure 4D.) Figure 6A_original.tif. (Original unmanipulated image used for Figure 6A.) Figure 6B_original.tif. (Original unmanipulated image used for Figure 6B.) Figure 6C_original.tif. (Original unmanipulated image used for Figure 6C.) Figure 6D_original.tif. (Original unmanipulated image used for Figure 6D.) Figure 7A_original.tif. (Original unmanipulated image used for Figure 7A.) Figure 7B_original.tif. (Original unmanipulated image used for Figure 7B.) Figure 7C_original.tif. (Original unmanipulated image used for Figure 7C.) Figure 7D_original.tif. (Original unmanipulated image used for Figure 7D and Figure S1A.) Figure S1A_original.tif. (Original unmanipulated image used for Figure S1A.) Figure S2.png. (Directionality histograms of the fibers under the cells from Figure 6B and D) Figure S3.png. (Fluorescence microscopy image of Schwann cells.) Figure S3A_original.tif. (Original unmanipulated image of Figure S3A.) Figure S3B_original.tif. (Original unmanipulated image of Figure S3B.) Figure S3C_original.tif. (Original unmanipulated image of Figure S3C.) Figure S4.tif. (Original unmanipulated SEM image of aligned Schwann cells on aligned nanofibers without PLL coating.) Zenodo: Repository for the Method Article "Oriented artificial nanofibers and laser induced periodic surface structures as substrates for Schwann cells alignment". https://doi.org/10.5281/zenodo.13944609 ( Lifka *et al.*, 2024). This project contains the following extended data: Bearing bracket Fixed bearing.step. (CAD .step file for the fixed bearing bracket of the rotating drum collector setup.) Bearing bracket loose fit bearing.step. (CAD .step file for the loose bearing bracket of the rotating drum collector setup.) Bearing cover Fixed bearing.step. (CAD .step file for the fixed bearing cover of the rotating drum collector setup.) Bearing cover loose fit bearing.step. (CAD .step file for the loose bearing cover of the rotating drum collector setup.) Motor mount.step. (CAD .step file for the DC motor mount of the rotating drum collector setup.) Surface structured collector.step. (CAD .step file for the surface-structured rotating drum collector.) Surface Structured Collector Drawing.pdf. (2D drawing of the rotating drum collector.) Setup Rotating Collector.pdf. (2D drawing of the whole rotating drum collector setup.) Non-Woven_Detachement_from_Collector.mkv (Short video showing the detachment process of the non-woven fabric from the surface-structured collector.) Data are available under the terms of the Creative Commons Attribution 4.0 International (CC-BY 4.0).
