# Supplementary material for: Oriented artificial nanofibers and laser induced periodic surface structures as substrates for Schwann cells alignment
Source: Open Res Eur. 2024 Oct 22;4:80. Originally published 2024 Apr 24. [Version 3] doi: 10.12688/openreseurope.17370.3 (PMC11541076; doi:10.12688/openreseurope.17370.3)
Supplement: Peer review history

# Reviewer report: Reviewer response for version 3

**DOI:** 10.21956/openreseurope.20321.r45362

**Published:** 2024-11-06

**Authors:** Tak-Ho Chu

**Affiliations:** 1 University of Calgary, Calgary, Canada

I reviewed the article carefully, and the authors have satisfactorily addressed all my concerns. I am pleased to approve the manuscript; however, I suggest that the authors revise the discussion for greater coherence.

Is the rationale for developing the new method (or application) clearly explained?

Partly

Is the description of the method technically sound?

Partly

Are the conclusions about the method and its performance adequately supported by the findings presented in the article?

Partly

If any results are presented, are all the source data underlying the results available to ensure full reproducibility?

Yes

Are sufficient details provided to allow replication of the method development and its use by others?

Partly

Reviewer Expertise:

Peripheral nerve injury and repair, Schwann cell biology

I confirm that I have read this submission and believe that I have an appropriate level of expertise to confirm that it is of an acceptable scientific standard.

# Reviewer report: Reviewer response for version 2

**DOI:** 10.21956/openreseurope.19906.r44238

**Published:** 2024-09-19

**Authors:** Tak-Ho Chu

**Affiliations:** 1 University of Calgary, Calgary, Canada

The manuscript by Lifka et al. investigates an improved method for generating aligned electro spun fibres and laser-induced nanoripples on PET foils to promote Schwann cell alignment in nerve guide materials. The authors employed scanning electron microscopy to examine an immortalized mouse Schwann cell line on these materials and concluded that both methods allow Schwann cells to align parallel to the direction of the fibers and nanoripples.

 While the manuscript provides sufficient evidence of Schwann cell alignment, several methodologies are lacking that are crucial for fully evaluating the study. My major concern is that the authors state in the Discussion that nanoripple formation on stiff materials limits its application (if I understand correctly), which is why electro spun fibres were investigated. Firstly, the order of these two experiments is reversed in the Results section. Secondly, it is neither shown nor discussed whether laser-induced nanoripples can form on softer materials. Therefore, the rationale behind the experiments needs further clarification and should be explicitly stated in the Introduction. Additionally, a brief literature review should be provided to outline the current development of nerve guides, their associated challenges, and what the authors aim to advance in the field.

 The manuscript would be significantly improved if the authors could address the following points:

1. Is PLL coating required for cell attachment to the electro spun fibres? If not, an experiment without PLL coating should be included. The authors should also discuss the materials used: are they biocompatible? Do they trigger an inflammatory response? Are they biodegradable? If the focus is solely on the technique rather than the materials, please discuss how easily these techniques can be applied to appropriate materials in the future.
2. References on nerve regeneration are missing. Readers unfamiliar with this field may benefit from the following reviews: Jessen and Mirsky, *The Success and Failure of the Schwann Cell Response to Nerve Injury*, *Front. Cell. Neurosci.*, 2019, and Cattin et al., *Macrophage-Induced Blood Vessels Guide Schwann Cell-Mediated Regeneration of Peripheral Nerves*, *Cell*, 2015. Additionally, please provide references supporting the statement in the Introduction that Schwann cell orientation depends on ECM or the supporting material. Schwann cells tend to align in a parallel fashion in culture when near confluence.
3. Regarding LIPSS, please clarify why the particular laser settings were chosen and why they are suitable for Schwann cells.
4. The authors should discuss how these techniques could be applied in a 3D configuration for nerve conduits.
5. The staining method should be described, perhaps in supplementary data.
6. How many samples were used for data analysis?
7. Why was 8 m/s chosen for further study in cell seeding? Fibers generated at 14 m/s appear to have a narrower peak, suggesting better alignment than those produced at 8 m/s. Can the authors provide a statistical comparison between these methods, perhaps through the area under the curve? Similarly, is there a statistical difference between fiber diameters at different speeds? The implications of fiber diameter and how it affects cell kinetics should be discussed.
8. How was the directionality of cells determined? Was it based on the long axis of the cells?

Is the rationale for developing the new method (or application) clearly explained?

Partly

Is the description of the method technically sound?

Partly

Are the conclusions about the method and its performance adequately supported by the findings presented in the article?

Partly

If any results are presented, are all the source data underlying the results available to ensure full reproducibility?

Yes

Are sufficient details provided to allow replication of the method development and its use by others?

Partly

Reviewer Expertise:

Peripheral nerve injury and repair, Schwann cell biology

I confirm that I have read this submission and believe that I have an appropriate level of expertise to state that I do not consider it to be of an acceptable scientific standard, for reasons outlined above.

## References

[1] : The Success and Failure of the Schwann Cell Response to Nerve Injury. *Front Cell Neurosci* .2019;13: 10.3389/fncel.2019.00033 33 10.3389/fncel.2019.00033 30804758 PMC6378273

[2] : Macrophage-Induced Blood Vessels Guide Schwann Cell-Mediated Regeneration of Peripheral Nerves. *Cell* .2015;162(5) : 10.1016/j.cell.2015.07.021 1127-39 10.1016/j.cell.2015.07.021 26279190 PMC4553238

# Response

**Published:** 2024-10-18

**Authors:** Sebastian Lifka

**Affiliations:** Johannes Kepler University of Linz, Austria

First of all, we would like to thank the reviewer for the helpful comments and suggestions on our manuscript and it is with great pleasure that we upload a revised version. We can understand the concern of the reviewer. Therefore, we now explain that laser-induced nanoripples have also been observed in a number of other polymers. These polymers have different material properties to PET to a certain extent, but are all fairly inflexible as they are several 10 µm thick sheets, rigid plates or thin films on rigid substrates. They are therefore only suitable to a limited extent as nerve-conducting fillers in a flexible nerve regeneration conduit. There are a few approaches to produce laser-induced nanoripples on very thin flexible polymer films or on bendable polymer microfibers, but these materials are very delicate to handle. Therefore, an alternative method describes the production of a non-woven consisting of nanofibers. This non-woven is extremely flexible and can be adapted to many different shapes. It could be rolled up to fill a nerve regeneration conduit. These considerations (together with a number of new references) have been added partly to the Introduction and mainly to the Discussion section. Regarding the order of the experiments in the Results section. As the main focus is more on the nanofiber-method, this method is treated first in the Introduction, Materials, and Results section. In the Discussion, we start with the LIPSS method and conclude with the nanofiber method, partly for reasons of argumentation and partly for didactic reasons, as we would like to end with the main focus. Furthermore, we complemented the introduction with a brief literature review about state-of-the-art nerve guides. Please find below our detailed responses to the comments.

**Comment #1: **Is PLL coating required for cell attachment to the electro spun fibres? If not, an experiment without PLL coating should be included. The authors should also discuss the materials used: are they biocompatible? Do they trigger an inflammatory response? Are they biodegradable? If the focus is solely on the technique rather than the materials, please discuss how easily these techniques can be applied to appropriate materials in the future.

**Author response:** *Thank you very much for this question. PLL coating is not absolutely necessary for cell attachment, but we observed that cell attachment is much better with PLL coating. If the fibers are not coated, the samples have to be treated very carefully, otherwise the cells tend to detach. Thus, to ease the handling of the samples, we decided to perform PLL coating for every sample. We discussed this also in the manuscript, and added a SEM image (Figure S4) showing Schwann cells on an uncoated sample as a supplement to the Zenodo repository.* *Regarding the materials used, the PA6 used here for the nanofibers is not biocompatible. We are currently working on implementing this method for biocompatible materials such as CAB. PA6 currently works best with the setup used. In this work, we were primarily concerned with the method and less with the materials themselves. We have added a statement to the manuscript in which we discuss the transfer of this method to biocompatible materials.*  

**Comment #2:** References on nerve regeneration are missing. Readers unfamiliar with this field may benefit from the following reviews: Jessen and Mirsky, The Success and Failure of the Schwann Cell Response to Nerve Injury, Front. Cell. Neurosci., 2019, and Cattin et al., Macrophage-Induced Blood Vessels Guide Schwann Cell-Mediated Regeneration of Peripheral Nerves, Cell, 2015. Additionally, please provide references supporting the statement in the Introduction that Schwann cell orientation depends on ECM or the supporting material. Schwann cells tend to align in a parallel fashion in culture when near confluence.

**Author response:** *Thank you very much for providing us with these beneficial references, we added them in the introduction section. Furthermore, we provided references supporting the statement in the Introduction that Schwann cell orientation depends on ECM or the supporting material (Jiang et al., 2024; Manganas et al., 2022).*  

**Comment #3:** Regarding LIPSS, please clarify why the particular laser settings were chosen and why they are suitable for Schwann cells.

**​​​​​​​Author response:** *These parameters were investigated in previous works (Barb et al., 2014, Barb et al., 2015, and Peterbauer et al., 2010). In the parameter range where “surface-induced alignment” appears, no qualitative difference of the alignment effect could be observed. However, we expect no strong qualitative effect of a variation of the structure size (within this regime) on the alignment. We discussed this in the discussion section.*  

**Comment #4:** The authors should discuss how these techniques could be applied in a 3D configuration for nerve conduits.

**​​​​​​​Author response:** *As stated above, the LIPSS on PET-foils are rather inflexible and therefore are only suitable to a limited extent as nerve guiding fillers in a bendable nerve regeneration conduit, but may be used as a hollow tube connecting two nerve ends in cases where extremely high flexibility is not absolutely necessary. Thus, we describe an alternative method using flexible, aligned nanofibers. These very thin nanofiber sheets can easily be rolled up in the axial direction of the fibers. This results in a thin roll of nanofiber non-woven with the nanofiber orientation in axial direction. One or more of these rolls could now be used as a conduit for nerve regeneration. Additionally, a thin laser-structured polymer foil can be wrapped around the nanofiber conduit in order to increase the stability of the whole conduit.* *To make this point clear, we described this intended process in the manuscript.*  

**Comment #5:** The staining method should be described, perhaps in supplementary data.

**​​​​​​​Author response:** *As requested, we added a brief description of the staining method in the supplementary data.*  

**Comment #6:** How many samples were used for data analysis?

**​​​​​​​Author response:** *In total, n = 7 samples for cells on aligned nanofibers where investigated. The here presented data show the typical results of one specific sample. We clarified this in the manuscript. As the method with LIPSS on PET foils was basically already investigated and the functionality could be shown for other types of cells in previous works (Peterbauer, 2008), the sample size of the LIPSS was n = 1. Only one sample was examined here to verify that this method also works for Schwann cells. As can be seen from the manuscript, this was also confirmed.*  

**Comment #7:** Why was 8 m/s chosen for further study in cell seeding? Fibers generated at 14 m/s appear to have a narrower peak, suggesting better alignment than those produced at 8 m/s. Can the authors provide a statistical comparison between these methods, perhaps through the area under the curve? Similarly, is there a statistical difference between fiber diameters at different speeds? The implications of fiber diameter and how it affects cell kinetics should be discussed.

**​​​​​​​Author response:** *Thank you for this important question. From the directionality analysis of the 8 m/s and the 14 m/s sample, it can be concluded that the two samples are more or less equal in terms of directionality. To quantify this assertion, we added and discussed the respective σ* *values (i.e., the standard deviation) of the Gaussian-Fit-curves (dashed red line) in the histograms. The standard deviation of the 8 m/s sample is with 8° nearly identical as the standard deviation of the 14 m/s sample with 7°. Therefore, it can be concluded that 8 m/s are sufficient for the production of high-quality aligned fibers. Since it is not absolutely necessary to increase the rotation speed to 14 m/s and considering that 14 m/s is the absolute limit of our setup, we decided to perform the cell experiments with an 8 m/s sample. We have also discussed this in detail in the manuscript.* *It seems that the measured fiber diameter increases with the rotational speed. However, in this work we did not investigate this behavior in more detail. If you take a closer look at the shape of the fibers at higher speeds, it seems that the shape becomes more and more ribbon-like. The increase in diameter can therefore possibly also be attributed to flattening of the fibers. Investigations in this direction would certainly be relevant in future work. We have also discussed this in the manuscript.*  

**Comment #8:** How was the directionality of cells determined? Was it based on the long axis of the cells?

**​​​​​​​Author response:** *The directionality of the cells was determined with the same method used for the analysis of the fibers (i.e., a Fourier components analysis of the SEM image). Yes, the analysis was based on the long axis of the cells, which corresponds to 0° in the histograms. We clarified this in the manuscript.*

# Reviewer report: Reviewer response for version 2

**DOI:** 10.21956/openreseurope.19906.r43363

**Published:** 2024-08-21

**Authors:** Harald Luksch

**Affiliations:** 1 Technische Universität München, Weihenstephan, Germany

The authors have responded well to my comments, and added additional figures to indicate the alignment statistics of the fibers. In addition, the expansion of the introduction and the overall presentation has improved the manuscript. I see no further necessity for additional changes.

Is the rationale for developing the new method (or application) clearly explained?

Yes

Is the description of the method technically sound?

Yes

Are the conclusions about the method and its performance adequately supported by the findings presented in the article?

Partly

If any results are presented, are all the source data underlying the results available to ensure full reproducibility?

Yes

Are sufficient details provided to allow replication of the method development and its use by others?

Yes

Reviewer Expertise:

Neuroscience, in-vitro studies of nervous system, neurophysiology, neuroanatomy.

I confirm that I have read this submission and believe that I have an appropriate level of expertise to confirm that it is of an acceptable scientific standard.

# Reviewer report: Reviewer response for version 1

**DOI:** 10.21956/openreseurope.18773.r40815

**Published:** 2024-06-27

**Authors:** Sandra I. Vieira, Nathalie Barroca

**Affiliations:** 1 Institute of Biomedicine (iBiMED) research unit - Department of Medical Sciences, University of Aveiro, Aveiro, Portugal; 2 TEMA research unit - Department of Mechanic Engineering, University of Aveiro, Aveiro, Aveiro, Portugal

Lifka et al. here present two methods for structuring surfaces aiming to improve the alignment of cells like Schwann cells, important for aiding the oriented growth of neuronal axons during peripheral nervous system regeneration. The methods are generally sound and the Zenodo repository associated with this article has examples of various of the steps taken and calculations performed. The authors cultured mouse Schwann cells for one week on top of the aligned nanofibers and PET nanoripples, which appear to have induced cell alignment. It is particularly visible the influence of the nanorippled topography as an external stimulus that renders the Schwann cells’ alignment.

 The Introduction section has some relevant reported data, but nothing on the biological problem that authors aim to tackle, which should not only be addressed in the Abstract (attention that PNI regeneration is not that ineffective – only in ‘large defects’). To better contextualize the need for the here-developed biomaterials, authors should include a short characterization of the PNS regeneration process, including the role of Schwann cells in bands of Bungner, in the Introduction section. This is necessary to support the author’s Conclusion that the alignment of Schwann cells may subsequently lead to better axonal regeneration. As for the manufacturing technologies, the introduction should also contextualize better the current state of the art regarding the exploitation of electrospinning and LIPSS, to highlight their relevance for repairing strategies for the PNS. For instance, the direct laser patterning technique has been already used to fabricate nanoripples and dual-rough hierarchical micro/nano structures to control Schwann cells attachment and migration. [Refer ref 1].

 Also, since nano-structuring is known for both boosting and inhibiting cell functions, this should be carefully explained in the introduction. 

 One of the methods here described applied electrospinning to create aligned polyamide nanofibers, in which a fast-rotating structured collector with a knurled surface is used, to allow the nanofibers to be effectively peeled off. Regarding the cellular alignment assay, one thing noticed is that the nanofibers of Figure 6B augmentation do not seem aligned, and authors should present a more representative zoom-in. Also, no control such as randomly aligned nanofibers, was here included for comparison purposes, and so the readers cannot visually certify the cell alignment effect. As this biomaterial is the core of this article, it would also be desirable to a) apply the same strategy as the one used in Figure 5 to have some quantitative data on cell alignment; b) perform a fluorescence microscopy analysis of e.g. the actin fibers of the cells, to complement the SEM assay.

 The other method here described used a laser beam to produce grooves on the surface of a flat PET foil. It would be important that the authors showed the PET nanoripples produced by the laser beam without the presence of cells (as they did for the nanofibers in Figure 4). As nano-structuring can either promote or inhibit cell functions, nanoripples should be carefully characterized (by AFM for instance) to give readers a quantified indication of the nanostructure quality and period that promoted Schwann cells orientation. Optimum LIPSS formation should be identified. The LIPSS as a response to the laser parameters should be thoroughly explored. For instance, LIPSS in polymers can depend on the energy distribution within pulses, polarization direction, and wavelength as well as repetition rate, therefore influencing both the period and quality of the resulting nanostructures. If the authors are unable to thoroughly explore laser parameters to optimize the LIPSS formation, they should at least explain in the discussion section how they selected the current parameters and how their variation would be expected to affect the nanoripples formation. 

 Other issues that in our opinion should be addressed to improve the article:

 - Schwann cells cultures (better subtitle) should include the cell density upon seeding on the coated biomaterials.

 - The biological readout is a single one (visual analyses of SEM images) and the article would gain with other host-interaction tests, such as cell viability, proliferation and/or adhesion

 -The Discussion is scarce and would gain with examples of other reported aligned nanofibers using different polymers and cues for neural cells, and by hypothesizing how could the biomaterials here presented be employed in in vivo strategies.

Is the rationale for developing the new method (or application) clearly explained?

Partly

Is the description of the method technically sound?

Yes

Are the conclusions about the method and its performance adequately supported by the findings presented in the article?

Partly

If any results are presented, are all the source data underlying the results available to ensure full reproducibility?

Yes

Are sufficient details provided to allow replication of the method development and its use by others?

Yes

Reviewer Expertise:

Sandra I. Vieira - Neuroscience, neuroregeneration and neurodegeneration, in-vitro studies of nervous system, host-biomaterials interactions.  Nathalie Barroca – manufacturing of platforms for bone and neural tissue repair; electromechanical couplings in biomaterials and tissues

We confirm that we have read this submission and believe that we have an appropriate level of expertise to confirm that it is of an acceptable scientific standard, however we have significant reservations, as outlined above.

## References

[1] : Cell patterning via laser micro/nano structured silicon surfaces. *Biofabrication* .2017;9(2) : 10.1088/1758-5090/aa71c6 025024 10.1088/1758-5090/aa71c6 28485302

# Response

**Published:** 2024-08-14

**Authors:** Sebastian Lifka

**Affiliations:** Johannes Kepler University of Linz, Austria

We would like to thank the reviewer for the helpful comments and suggestions on our manuscript and it is with great pleasure that we upload a revised version. To improve the quality of our paper, we revised our manuscript according to the reviewers’ suggestions for improvement. Below is our detailed response to the reviewers' comments.

**Comment #1:** *The Introduction section has some relevant reported data, but nothing on the biological problem that authors aim to tackle, which should not only be addressed in the Abstract (attention that PNI regeneration is not that ineffective – only in ‘large defects’). To better contextualize the need for the here-developed biomaterials, authors should include a short characterization of the PNS regeneration process, including the role of Schwann cells in bands of Bungner, in the Introduction section. This is necessary to support the author’s Conclusion that the alignment of Schwann cells may subsequently lead to better axonal regeneration.* In the peripheral nervous system of vertebrates, neurons are responsible for receiving and transmitting electrical and chemical signals and glial cells provide the necessary support and protection for neurons. Here, the most important glial cells are Schwann cells. They are responsible for the creation of myelin sheaths that protect and insulate neuronal axons. They are also essential and indispensable for axon regeneration in the event of injury. The peripheral axons regenerate in regeneration tracks (i.e., Bünger bands) formed by elongated Schwann cells. Small gaps can regenerate from alone while large gaps are considered to require the support of a nerve graft. But the currently proposed artificial nerve grafts have insufficient success rates, especially for large nerve gaps of several cm, and nerve autografts cause severe functional loss or risk of morbidity at the donor sites. A key factor here is the elongated orientation of the Schwann cells which is induced by stimuli from their surrounding either in the extra cellular matrix or in case of artificial implants by the supporting material. We have added these considerations in a new paragraph at the beginning of the section Introduction with the new reference Manganas et al., 2022.

**Comment #2:** *As for the manufacturing technologies, the introduction should also contextualize better the current state of the art regarding the exploitation of electrospinning and LIPSS, to highlight their relevance for repairing strategies for the PNS. For instance, the direct laser patterning technique has been already used to fabricate nanoripples and dual-rough hierarchical micro/nano structures to control Schwann cells attachment and migration. [Refer ref 1]. Also, since nano-structuring is known for both boosting and inhibiting cell functions, this should be carefully explained in the introduction.*

 We have added two new paragraphs in the section Introduction. i.e., the second and the third, with more details on the state of the art regarding the exploitation of electrospinning and LIPSS for nerve repair graft and and control of cell growth, respectively. We refer here to the new references (Chen at., 2021; Wang et al., 2018; Kriebel et al., 2017; Wang et al., 2020; Fosodeder et al., 2020; Muck et al., 2021; Maalouf et al., 2022), as well as the reference (Yiannakou et al., 2017), which was suggested by the reviewer.  

**Comment #3:** *Regarding the cellular alignment assay, one thing noticed is that the nanofibers of Figure 6B augmentation do not seem aligned, and authors should present a more representative zoom-in.*

 Thank you very much for bringing this to our attention. We have added two new SEM images which show the underlying nanofibers in more detail. Figure 6B now shows random oriented nanofibers under the cells and Figure 6D shows the aligned nanofibers under the cells. Additionally, we performed a directionality analysis of the nanofibers under the cells. The resulting histograms show a clear orientation of the nanofibers and can be found in the Zenodo repository as extended Data (Figure S2).  

**Comment #4:** *Also, no control such as randomly aligned nanofibers, was here included for comparison purposes, and so the readers cannot visually certify the cell alignment effect. As this biomaterial is the core of this article, it would also be desirable to a) apply the same strategy as the one used in Figure 5 to have some quantitative data on cell alignment; b) perform a fluorescence microscopy analysis of e.g. the actin fibers of the cells, to complement the SEM assay.*

 As already requested by Reviewer 1, we applied the same analysis methods as for the oriented nanofibers to the cells and included the resulting histograms in the manuscript. In addition, we performed fluorescence microscopy of cells on nanofibers (on flat cover glass surface, randomly oriented, and aligned) and on a flat cover glass as control as requested. For space reasons, these images are only available in the Zenodo repository as extended data (Figure S3). The reason why we originally only analyzed SEM images was because this is the gold standard for analyzing the directionality of fibers or cells. On the other hand, the underlying fibers are not visible in fluorescence microscopy and thus it cannot be verified and shown that the cells really spread in the fiber direction.  

**Comment #5:** *The other method here described used a laser beam to produce grooves on the surface of a flat PET foil. It would be important that the authors showed the PET nanoripples produced by the laser beam without the presence of cells (as they did for the nanofibers in Figure 4).*

 We replaced one zoom-in SEM image of Figure 7 with an even more zoomed-in SEM image of the nanoripples without cells.  

**Comment #6:** *As nano-structuring can either promote or inhibit cell functions, nanoripples should be carefully characterized (by AFM for instance) to give readers a quantified indication of the nanostructure quality and period that promoted Schwann cells orientation.*

 We absolutely agree with the reviewer. These nanoripples have already been characterized in our previous work (Buchberger et al., 2023 and Heitz et al., 2024) using optical methods and AFM. The typical spatial period is about 330 nm and the height is between 110 nm and 160 nm. We have described this in more detail in the manuscript and referred to the two papers mentioned.  

**Comment #7:** *Optimum LIPSS formation should be identified. The LIPSS as a response to the laser parameters should be thoroughly explored. For instance, LIPSS in polymers can depend on the energy distribution within pulses, polarization direction, and wavelength as well as repetition rate, therefore influencing both the period and quality of the resulting nanostructures. If the authors are unable to thoroughly explore laser parameters to optimize the LIPSS formation, they should at least explain in the discussion section how they selected the current parameters and how their variation would be expected to affect the nanoripples formation.*

 In our previous work, we have investigated (for other cell types) the parameter range within one can observe “surface-induce alignment” of the cells. Within this range we saw no qualitative difference of the alignment effect. Both methods presented here provide parallel features which correspond to surface-induced alignment regime and we show here for the first time that they can be used for the orientation of Schwann cells, in deed. However, we expect no strong qualitative effect of a variation the structure size (within this regime) on the alignment. We have added these considerations in a new paragraph at the end of the section Discussion with the new references Barb et al., 2014, Barb et al., 2015, and Peterbauer et al., 2010.  

**Comment #8:** *Schwann cells cultures (better subtitle) should include the cell density upon seeding on the coated biomaterials.*

 The cell density upon seeding was 25,000 cells per cm2 resulting in a total number of approximately 65,000 cells per sample. We added this to the cell cultivation subsection in the manuscript.  

**Comment #9:** *The biological readout is a single one (visual analyses of SEM images) and the article would gain with other host-interaction tests, such as cell viability, proliferation and/or adhesion.*

 We can understand the reviewer's view, but we are currently still struggling with our methods to determine cell adhesion, as the samples used here are very flexible and therefore cannot be measured reliably with our methods. Furthermore, when determining viability and proliferation, we see the problem that our samples are structured and therefore have a significantly different surface size than a flat control sample, which in our opinion makes the evaluation difficult and not very meaningful. A single cell analysis might be possible, but it is difficult and extremely imprecise. As this article focuses on a method for directional growth of Schwann cells, we have refrained from determining additional parameters.  

**Comment #10:** *The Discussion is scarce and would gain with examples of other reported aligned nanofibers using different polymers and cues for neural cells, and by hypothesizing how could the biomaterials here presented be employed in in vivo strategies.*

 We see the point of the reviewer, but we think we have addressed it already in the description of the state of the art in the second paragraph of the section Introduction (see above).

# Reviewer report: Reviewer response for version 1

**DOI:** 10.21956/openreseurope.18773.r40812

**Published:** 2024-06-08

**Authors:** Harald Luksch

**Affiliations:** 1 Technische Universität München, Weihenstephan, Germany

In their submission entitled “Oriented artificial nanofibers and laser induced periodic surface structures as substrates for Schwann cells alignment”, Lifka and colleagues describe a way of structuring surfaces for better orientation of biological cells. Such orientation might be helpful for oriented growth of neurons in, e.g, regeneration processes after injuries to the peripheral nervous system.

 Lifka et al use two different methods for structuring the surface, namely the production of grooves with a laser beam, or the production of a nanofiber mat with electrospun polyamide nanofibers. For the latter process, a key feature of the spinning process was improved by using a fast rotating structured collector with a knurled surface. The woven nanofiber mat can be removed without damage or residual material attached to the collector. Both the mat and the laser-fabricated grooves were inspected with Scanning Electron Microscopy and showed well aligned fibers or grooves,  respectively.

 The authors then cultivated mouse Schwann cells from a cell line onto either substrate for a week, and analysed the orientation of the cells and their processes. From the images provides in the submission, a clear influence of the structure on the cellular orientation is evident. However, a quantitative assessment of cell orientation in both the control cultures and the cultures on structured surfaces would have been good, to clearly show the orientation and to quantify the effect of the given structure. After all, the parameters chosen for either structuring method might be suboptimal, and variations of the structures (i.e., deeper or wider grooves etc.) might lead to even better orientation control. The authors conclude that their production of mechanical nanofeatures is capable of influencing glial cells into an oriented growth, and thus may potentially aid axonal regeneration.

 My impression is that this report is a valuable addition to manufacturing processes for structured surfaces for nerve regeneration. The method description is sound, the images illustrate the respective issues well, and the conclusions are well founded.

Is the rationale for developing the new method (or application) clearly explained?

Yes

Is the description of the method technically sound?

Yes

Are the conclusions about the method and its performance adequately supported by the findings presented in the article?

Partly

If any results are presented, are all the source data underlying the results available to ensure full reproducibility?

Yes

Are sufficient details provided to allow replication of the method development and its use by others?

Yes

Reviewer Expertise:

Neuroscience, in-vitro studies of nervous system, neurophysiology, neuroanatomy.

I confirm that I have read this submission and believe that I have an appropriate level of expertise to confirm that it is of an acceptable scientific standard, however I have significant reservations, as outlined above.

# Response

**Published:** 2024-08-14

**Authors:** Sebastian Lifka

**Affiliations:** Johannes Kepler University of Linz, Austria

We would like to thank the reviewer for the helpful comments and suggestions on our manuscript and it is with great pleasure that we upload a revised version. To improve the quality of our paper, we revised our manuscript according to the reviewers’ suggestions for improvement. Below is our detailed response to the reviewers' comments.

**Comment #1:** *From the images provides in the submission, a clear influence of the structure on the cellular orientation is evident. However, a quantitative assessment of cell orientation in both the control cultures and the cultures on structured surfaces would have been good, to clearly show the orientation and to quantify the effect of the given structure.*

 Thank you very much for that valuable input, we added a figure (Figure 8) where we analyzed the directionality of the cells on the nanofibers (random oriented and aligned) and on the PET foils (flat and with nanoripples) using the same method as for the analysis of the aligned nanofibers. The resulting histograms show a clear orientation of the cells on aligned nanofibers and nano-structured PET foils.

**Comment #2:** *After all, the parameters chosen for either structuring method might be suboptimal, and variations of the structures (i.e., deeper or wider grooves etc.) might lead to even better orientation control.* 

 In our previous work, we have investigated (for other cell types) the parameter range within one can observe “surface-induce alignment” of the cells. Within this range we saw no qualitative difference of the alignment effect. Both methods presented here provide parallel features which correspond to surface-induced alignment regime and we show here for the first time that they can be used for the orientation of Schwann cells, in deed. However, we expect no strong qualitative effect of a variation the structure size (within this regime) on the alignment. We have added these considerations in a new paragraph at the end of the section Discussion with the new references Barb et al., 2014, Barb et al., 2015, and Peterbauer et al., 2010.
